# Validity of accuracy and trending ability of non-invasive continuous total hemoglobin measurement in complex spine surgery: a prospective cohort study

**DOI:** 10.1186/s12871-019-0790-y

**Published:** 2019-07-04

**Authors:** Feng-Cheng Chang, Jr-Rung Lin, Fu-Chao Liu

**Affiliations:** 1 Department of Anesthesiology, Chang Gung Memorial Hospital, Linkou Medical Center, Chang Gung University, No. 5, Fusing St, Guishan District, Taoyuan City, 33305 Taiwan; 2 Graduate Institute of Clinical Medical Sciences, College of Medicine, Chang Gung University, Taoyuan, Taiwan; 3 Clinical Informatics and Medical Statistics Research Center and Graduate Institute of Clinical Medicine, Chang Gung University, Taoyuan, Taiwan; 4 College of Medicine, Chang Gung University, Taoyuan, Taiwan

**Keywords:** Non-invasive, Hemoglobin, Monitor, Pulse CO-oximeter, SpHb, Radical-7, Spine surgery

## Abstract

**Background:**

Patients undergoing complex spine surgery present with multilevel spinal involvement, advanced age, and multiple comorbidities. Surgery is associated with significant blood loss and remarkable hemodynamic changes. The present study aimed to investigate the accuracy and trending ability of a non-invasive continuous method to monitor hemoglobin (SpHb) concentrations using a Radical-7™ Pulse CO-Oximeter in complex spine surgery.

**Methods:**

Forty-nine patients who underwent complex spine surgery were enrolled in this prospective observational study. Multiple time points were established for data collection throughout the operation. Simultaneous SpHb–total hemoglobin (tHb) paired data were recorded for analyses. Linear regression analysis, Bland–Altman plot, four-quadrant plot, and Critchley polar plot were used to assess the accuracy and trending ability of the monitor.

**Results:**

A total of 272 pairs of SpHb-tHb data were available and were divided into two groups based on the perfusion index (PI): PI values ≥1.0 (*n* = 200) and PI values < 1.0 (*n* = 72). The correction coefficients (r) between SpHb and tHb were 0.6946 and 0.6861 in the groups with PI values ≥1.0 and < 1.0, respectively (*P* < 0000.1). In the ≥1.0 group, the mean bias was − 0.21 g/dL and the percentage error (PE) was 15.85%, whereas in the < 1.0 group, the mean bias was − 0.04 g/dL and the PE was 17.42%. Four-quadrant plot revealed a concordance rate of 85.11%, whereas the Critchley polar plot showed a concordance rate of 67.21%.

**Conclusions:**

The present study demonstrates the acceptable accuracy of the Radical-7™ Pulse CO-Oximeter even with a low PI. However, the trending ability was limited and unsatisfactory.

## Background

Total hemoglobin (tHb) concentration continues to be the reference or threshold for administering packed red blood cells in clinical practice [1]. In some instances, patients may require adequate fluid supplements and transfusion during major surgical procedures owing to significant hemodynamic changes that are associated with blood loss. Adequate transfusions can be performed when accurate measurements of the tHb concentration are obtained using laboratory methods during the perioperative phase. The standard central laboratory method involving invasive blood sampling continues to be implemented. Although several operation rooms have adopted the point-of-care testing blood gas analysis in satellite laboratories, time-consuming and cost issue are still considerable. Moreover, the transfusion therapy strategy varies widely among the clinicians [2]. Furthermore, perioperative anemia is associated with poor outcomes following surgery and should be avoided as far as possible [3]. In addition, unnecessary blood transfusions may be related to adverse risks such as infection and transfusion-related lung injury as well as increase the length of hospitalization, thereby affecting the clinical outcome [4, 5].

The Masimo Radical-7™ Pulse CO-Oximeter (Masimo Corporation, Irvine, USA) is a commercially available device to monitor hemoglobin concentration, providing a real-time, continuous, non-invasive measurement during various major surgeries and in critical populations under intensive care. Using multi-wavelength spectrophotometric detection, this device analyzes the different optical densities passing through the tissue. In interpretation of the digital signal, the immediate and continuous hemoglobin (SpHb) concentration may become one of the parts of monitors and offer the physicians a reference point in decision-making.

Complex spine surgery procedures have increasingly been performed over the past few decades [6, 7], with a reported complication rate of 7% [8]. The population undergoing these types of complex procedures characteristically present with multilevel spinal involvement, advanced age, and multiple comorbidities [8–10]. Moreover, prolonged operation time and significant blood loss are important factors that contribute to instability in these patients during the surgical procedure. Although several studies have examined different surgical methods and in different populations [11–24], only few of these studies have determined the validity of SpHb in spine surgery [19, 20]. Ehrenfeld et al. have reported that the SpHb-guided blood management in various orthopedic surgeries was associated with a 4% absolute reduction in the risk of blood transfusion compared with the standard care without the monitoring of SpHb values. However, that study did not elaborate upon the accuracy of SpHb [21]. The present study aimed to validate the accuracy and trending ability of SpHb measured using Radical-7™ Pulse CO-Oximeter during major spine surgeries.

## Methods

This study was approved by the Institutional Review Board of Chang Gung Medical Foundation in Taiwan (registration number: 201701229B0). Informed consent was obtained from all the voluntary participants after the details of the study were explained by the study investigators. Overall, 50 patients scheduled to undergo spine surgery at the Chang Gung Memorial Hospital in Taiwan, between August 2017 and January 2018, were enrolled in this prospective study. Patients who were aged < 20 years; those who exhibited an inability to use the upper extremities for monitoring; those who were diagnosed with preoperative arrhythmia, peripheral vascular diseases, hematologic diseases; and those who refused to provide consent were excluded. The criteria for complex or complicated spine surgery were as follows: the involvement of ≥3 levels of the lumbar spine segment; estimated blood loss of > 500 mL or a prediction of blood transfusion; and an estimated period of ≥4 h for the complete procedure. Most patients underwent posterior spinal fusion with the instrumentation of implants. Indications for the surgical procedure included spinal stenosis, debridement, scoliosis correction, and instrumentation revision.

On arrival at the operation room, all patients were regularly monitored using a thermometer, electrocardiograph, pulse oximetry, and non-invasive blood pressure cuff. General anesthesia was performed in all patients using a standardized protocol. The induction anesthetics included propofol (1.5–2 mg/kg), fentanyl (1–2 mcg/kg), and cisatracurium (0.2 mg/kg) to facilitate tracheal intubation (6.5–7.5-mm oral cuffed tracheal tube). The mechanical ventilator was adjusted as follows: tidal volume, 6–8 mL/kg; respirator rate, 8–16 breaths per min; end-tidal CO_2_, 30–40 mmHg; and end expiratory pressure, 5 cmH_2_O. Following the induction of anesthesia and tracheal intubation, a 20-gauge catheter was implanted into the radial artery as an arterial line to monitor the perioperative blood pressure and to withdraw the blood sample for measurement of hemoglobin concentration. The adhesive sensor (R2 25, rev E) was wrapped onto the index finger of the patient and connected to the Radical-7™ Pulse CO-Oximeter to continuously monitor the SpHb value. After the completion of arterial line implantation, the blood pressure cuff was removed. To eliminate optical interference, the sensor was set to the hand without the arterial line, intravenous line, and pulse oximeter. The sensor was covered with opaque tape for protection from the ambient streams of light. The blood sample was analyzed in the operation room within 5 min after withdrawn from the radial arterial line. A blood gas analyzer (Stat Profile Critical Care Xpress, Nova Biomedical, Waltham, Mass, United States) was adopted in the operation room of our institution. The analyzer calibration was routinely performed according to the manufacturer’s instructions.

Several time points were established for data collection. The SpHb values were recorded for every blood sample (tHb) obtained, and the paired data were recorded during the following time points: immediately after the induction of anesthesia; at the start of the surgical procedure; every 1 hour thereafter; at the end of the operation or when the patient had arrived at the post-anesthesia room; and before leaving the post-anesthesia room. The anesthesiologists were blinded to the recorded data, and the study investigators were not involved in anesthesia management during the operation. The anesthesiologists were responsible for the perioperative fluid supplement and blood transfusion as well as for controlling the stability of the vital signs. The SpHb values from the Radical-7™ Pulse CO-Oximeter monitor did not influence the decision-making during the whole procedure.

Descriptive data including the basic patient demographic data, total surgery/anesthesia time and the number of spine levels involved were recorded. In addition, the amount of intravenous fluids, including crystalloids and blood products, were recorded.

### Statistical analysis

The demographic and clinical data were summarized as descriptive statistics. Patient characteristics are expressed as mean value ± standard deviation (SD). For analysis of the correlations between paired SpHb and tHb values, the linear regression analysis was applied. To assess the accuracy of SpHb, Bland–Altman analysis was performed, and the results were reported as the mean bias ±1.96 SD. The mean bias was calculated as the average difference between SpHb–tHb pairs. Limits of agreement (LoA) were calculated as the mean bias ±1.96 SD. The percentage error in this study was calculated with the LoA divided by the mean hemoglobin data {100 × [LOA/ (mean SpHb + mean tHb)/2]}. Although several studies have excluded data with low perfusion values [11, 23, 24], we sought to determine the accuracy in this group of paired data. Therefore, the present study, we compared the paired data with PI values < 1.0 with those having a PI value ≥1.0 to assess the accuracy of SpHb.

Trending ability was assessed in addition to the accuracy analysis. We used the four-quadrant plot and Critchley polar plot methods to test the magnitude and directionality of the change in hemoglobin concentration. The central exclusion zones were set as 1.0 (g/dL) in both statistical methods to minimize the interference of analysis. Following the exclusion of the data in the central zones, the concordance rate was defined as the proportion of the total number of the points in the same direction of changes in tHb and SpHb to the total number of points on the four-quadrant plot. In the Critchley polar plot, the concordance rate was defined as the percentage of points within the ±30° radial zone to the total number of points on the polar plot.

Statistical analysis was performed using the Statistical Package for the Social Sciences (SPSS) version 22.0 (SPSS, Chicago, Ill, United States) and R 3.2.0 statistical software (R Foundation for Statistical Computing, Vienna, Austria). A *P* value of < 0.05 was considered significant for all analyses.

## Results

Of the 50 patients initially enrolled in the study, one was excluded due to failure of the arterial line during the surgery, resulting in a total of 49 patients who were included in the final analysis. Patient demographic characteristics and perioperative data are shown in Table 1. Mean age of all patients (*n* = 49) was 65.53 ± 11.87 years. Based on the American Society of Anesthesiologists (ASA) classification, most cases were class 3 (85.7%), whereas the remaining were class 2 (14.3%). Estimated blood loss was 766.33 ± 564.71 mL (ranging from 100 to 2600 mL) and intravenous crystalloid supplement was 1474.49 ± 510.63 mL (450 to 3650 mL). The amount of blood products transfused during the surgery was as follows: packed red blood cells, 2.94 ± 2.35 units; and fresh frozen plasma, 2.33 ± 2.29 units. Mean surgical duration was 217.8 ± 68.4 min and the mean total anesthesia time was 264.43 ± 66.6 min. The number of spinal levels involved ranged from 3 to 13.

**Table 1:** The patient demographic characteristics and perioperative data

Characteristic (*N* = 49)	Descriptive statistics	Range
Age (years)	65.53 ± 11.87	21–85
Height (cm)	156.12 ± 9.23	139–177
Weight (kg)	64.64 ± 11.87	45–104
BMI (kg/m^2^)	26.51 ± 3.99	23.29–33.20
Preoperative baseline Hb (g/dL)	12.89 ± 1.58	8.6–16.6
IV crystalloid (mL)	1474.49 ± 510.63	450–3650
Blood loss (mL)	766.33 ± 564.71	100–2600
pRBC (units)	2.94 ± 2.35	0–8
FFP (units)	2.33 ± 2.29	0–8
Anesthesia time (minutes)	264.43 ± 66.6	160–591
Surgery time (minutes)	217.8 ± 68.4	115–566
Gender		
Male	17 (34.69%)	
Female	32 (65.31%)	
ASA classification		
Class 2	7 (14.29%)	
Class 3	42 (85.71)	
Total spine levels		
3	20 (41.67%)	
4	11 (22.92%)	
5	9 (18.75%)	
6	3 (6.25%)	
8	1 (2.08%)	
9	3 (6.25%)	
13	1 (2.08%)	

Data are described as mean ± standard deviation or number (%)

*BMI* body mass index, *Hb* hemoglobin, *IV* intravenous, *pRBC* packed red blood cells

*FFP* fresh frozen plasma, *ASA* American Society of Anesthesiologists

**Fig. 1:**
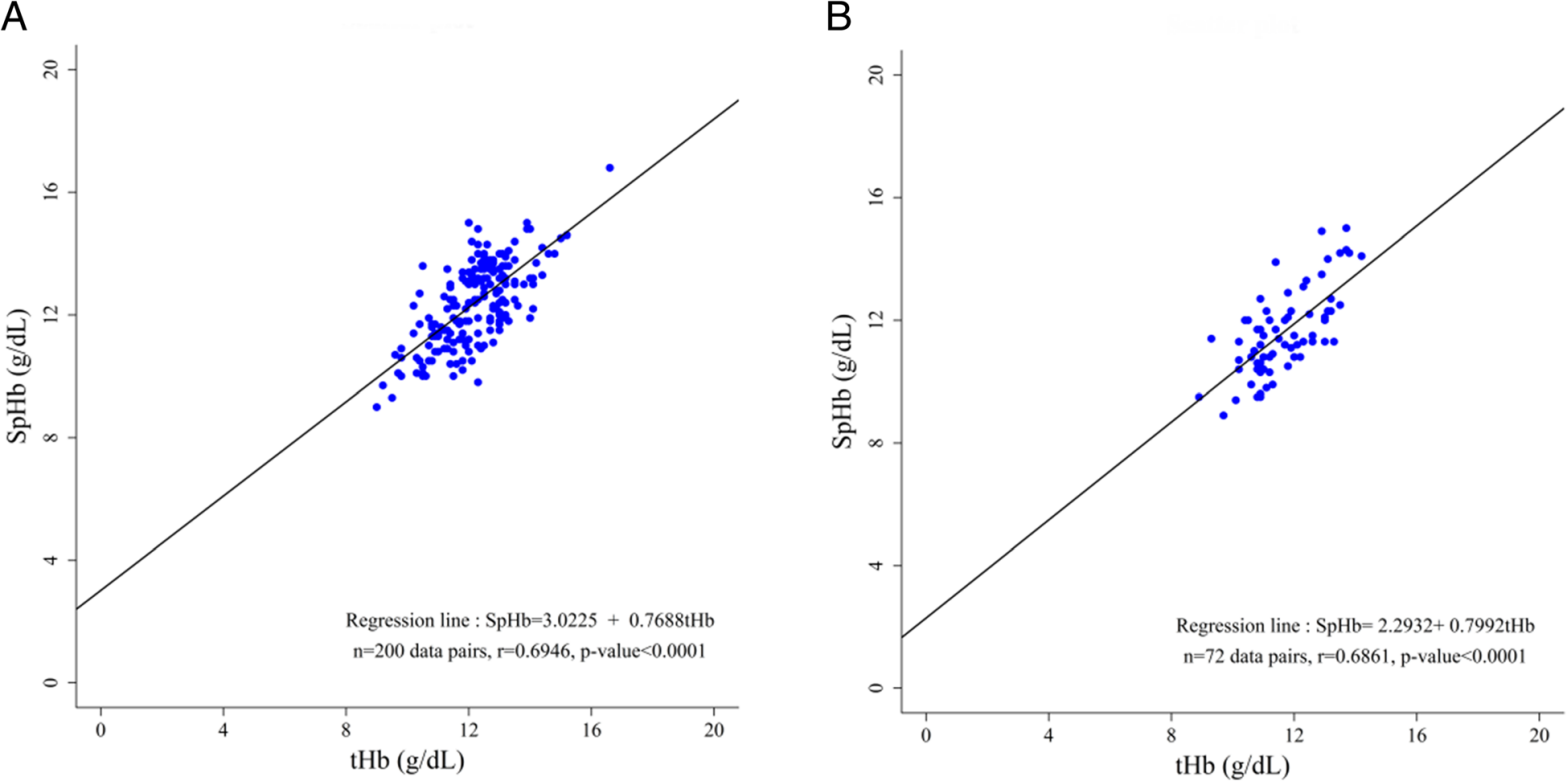
Linear regression analysis between SpHb and tHb. **a** *r* = 0.6946 in perfusion index values ≥1.0 group. **b** *r* = 0.6861 in perfusion index values < 1.0 group

Overall, 272 pairs of SpHb–tHb data were obtained and were divided into two groups based on the perfusion index (PI): PI values ≥1.0 (*n* = 200) and PI values < 1.0 (*n* = 72). The hemoglobin concentrations ranged from 8.9 to 16.8 g/dL (SpHb) and 9.3 to 16.6 g/dL (tHb). Linear regression analysis indicated correlations between SpHb and tHb values with a correction coefficient (r) of 0.6946 in the ≥1.0 group (Fig. 1a) and 0.6861 in the < 1.0 group (Fig. 1b) (both *P* < 0.0001).

**Fig. 2:**
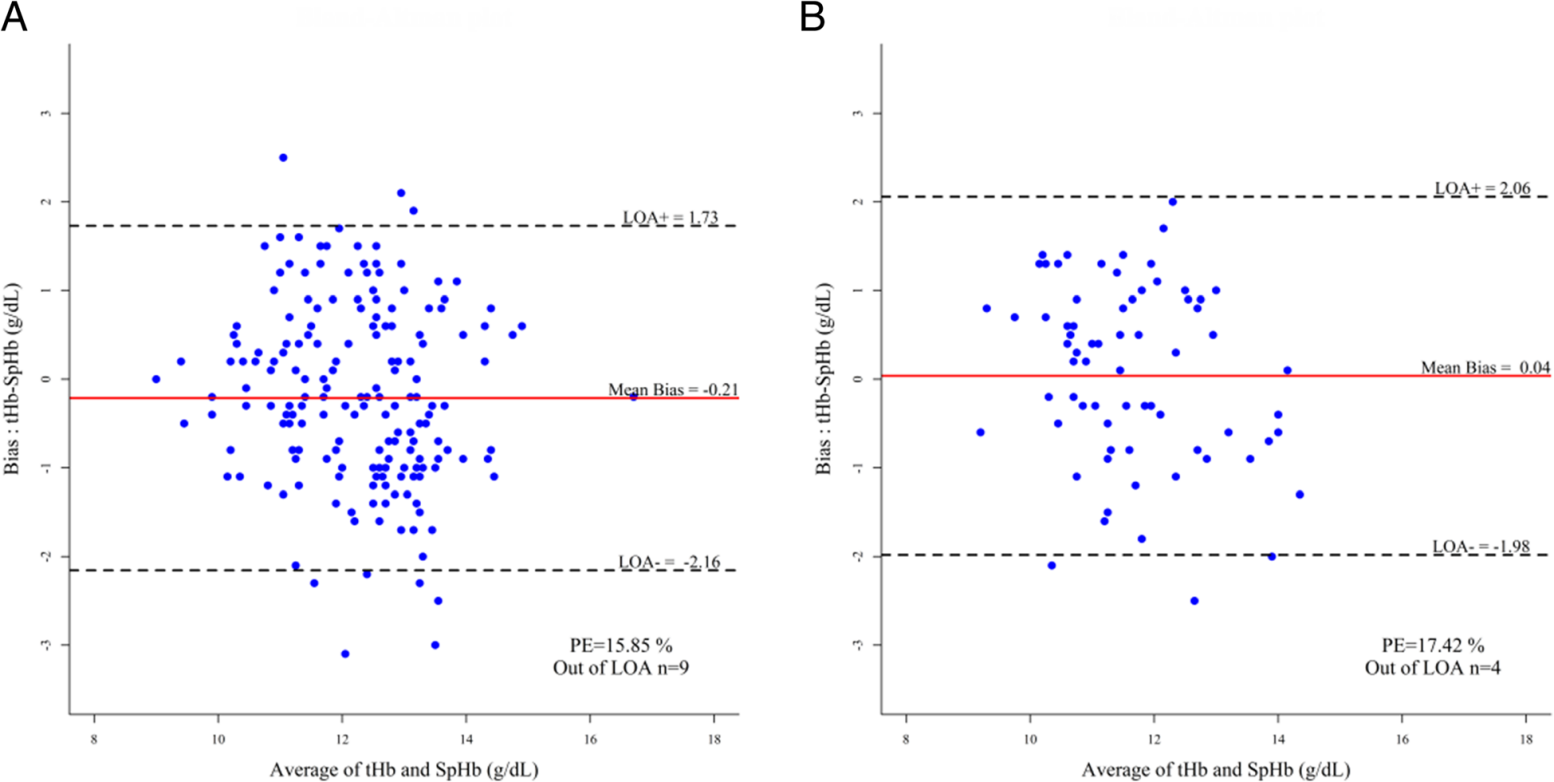
Bland-Altman analysis plots for SpHb and tHb. This plot shows mean bias and limits of agreement (±1.96 standard deviation) with the percentage error (%) and the number of out of limits of agreement. **a** 200 data pairs with perfusion index values ≥1.0 group. **b** 72 data pairs with perfusion index values < 1.0 group

The Bland–Altman analysis revealed the mean bias and LoA between SpHb and tHb with percentage errors. In the ≥1.0 group, the mean bias was − 0.21 g/dL with LoA ranging from − 2.16 to 1.73 g/dL and a percentage error of 15.85% (Fig. 2a). In the < 1.0 group, the mean bias was − 0.04 g/dL (LoA, − 1.98 to 2.06 g/dL; percentage error, 17.42%; Fig. 2b).

**Fig. 3:**
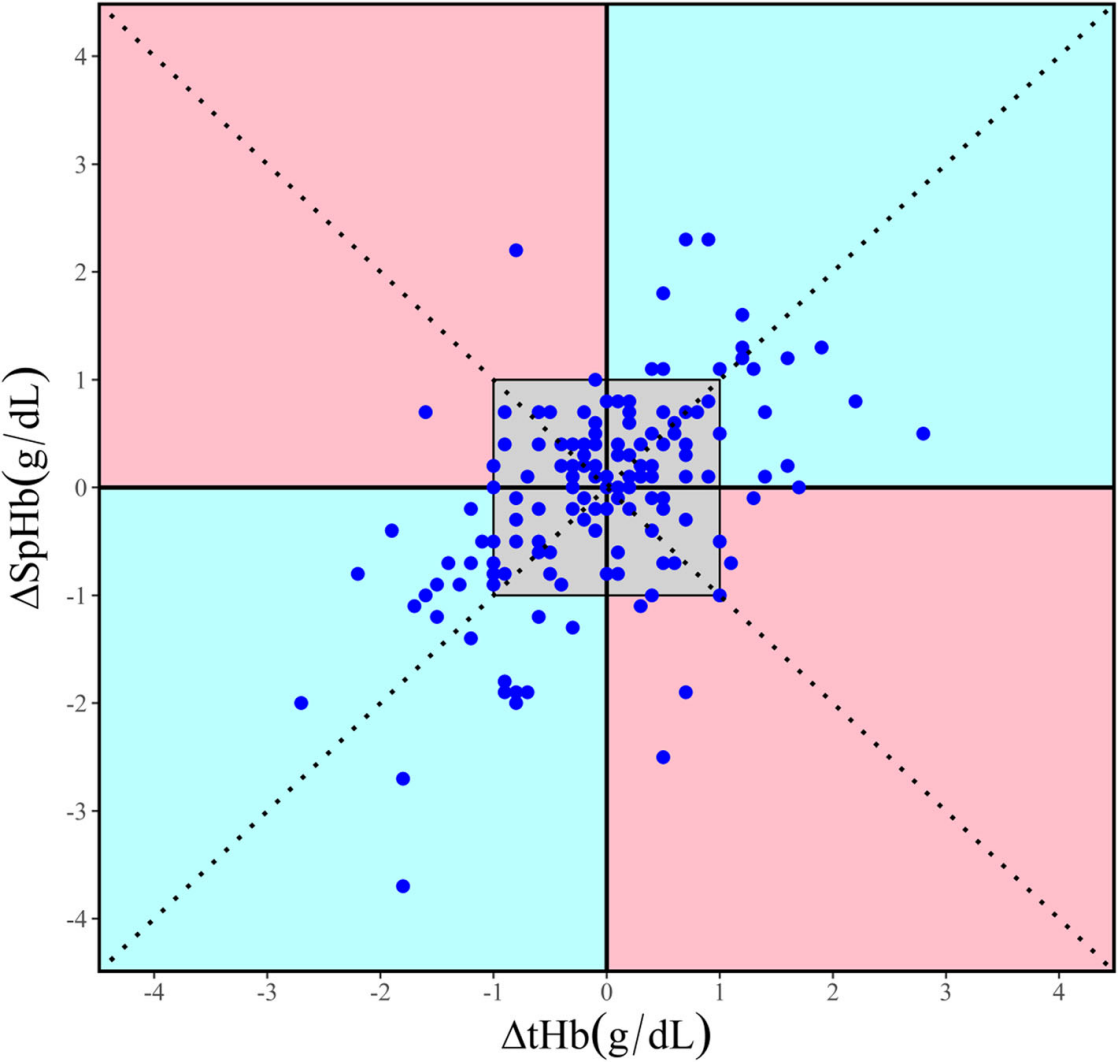
Four-quadrant plot compares the consecutive changes in SpHb and tHb. The central exclusion zone was applied at ±1.0 g/dL to rule out the pairs of data with minimal consecutive difference. The concordance rate was 85.11%

**Fig. 4:**
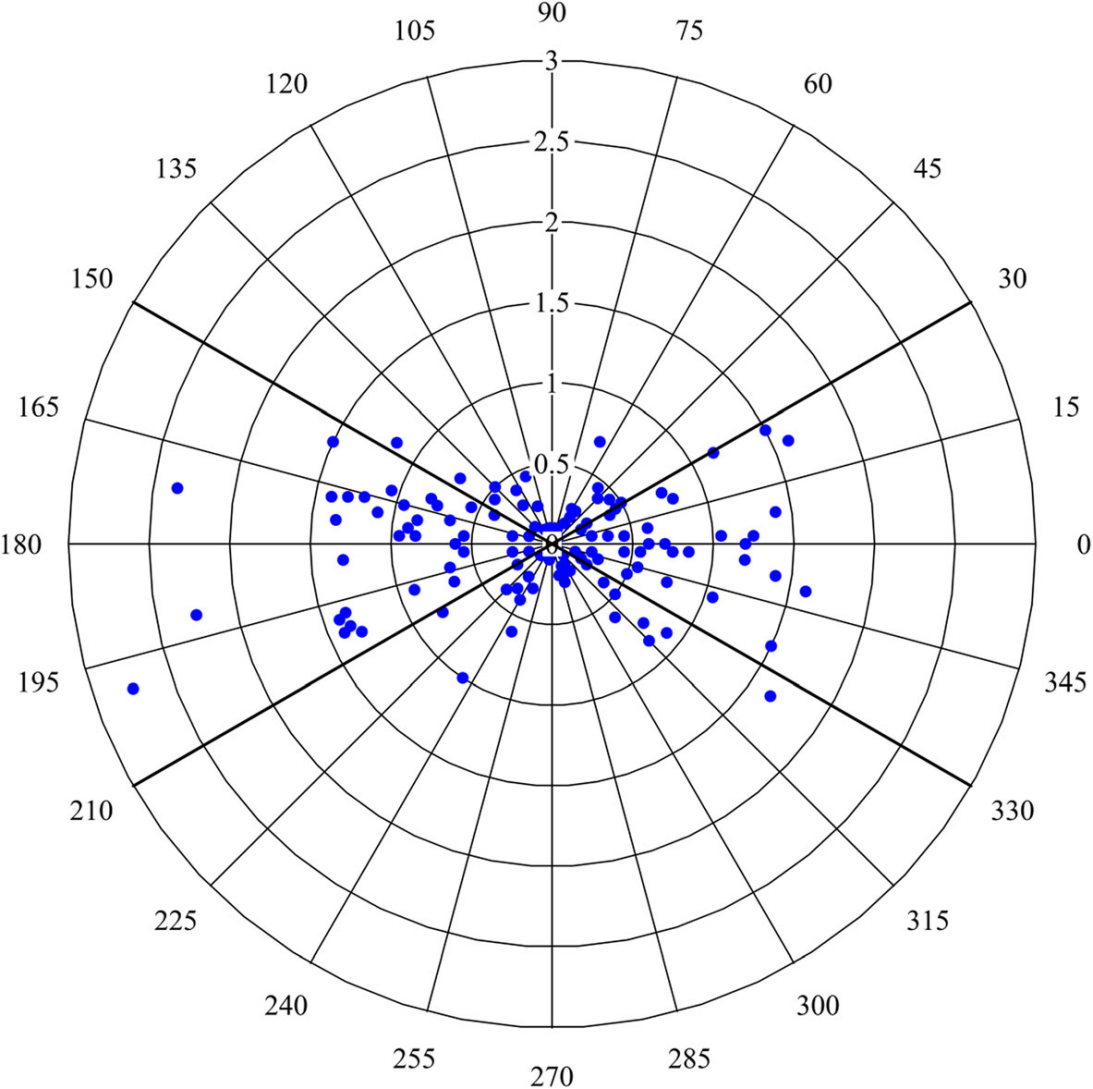
Critchley polar plot. The central exclusion zone was set at 1.0 g/dL as the radius. 67.21% of data was located in the limits of acceptable trending

To evaluate the trending ability, the 200 pairs of data with PI values ≥1.0 were placed on two plots (four-quadrant plot and Critchley polar plot). The central exclusion zone was applied at ±1.0 g/dL to rule out the pairs of data with minimal consecutive difference in both plots. The concordance rate was 85.11% in the four-quadrant plot (Fig. 3) and 67.21% in the Critchley polar plot (Fig. 4).

## Discussion

The accurate and rapid measurement of hemoglobin concentration is crucial during the decision-making process for fluid and transfusion therapy in complicated surgical procedures. Several articles have discussed the accuracy of Radical-7™ Pulse CO-Oximeter [11–18]; however, the lack of statistical agreement renders the clinical applicability of this method debatable. In our consideration, the ability of one device to provide helpful measurement data may prove valuable for monitoring during complicated surgical procedures as well as critical populations.

In the present study, we investigated patients undergoing complex spine surgery and presenting with the characteristic features of multilevel spinal involvement, prolonged operation time, significant blood loss, and remarkable hemodynamic changes. In addition, the intervention of fluid supplements, transfusion of blood products, and the use of vasopressors to perioperatively maintain the hemodynamic stability are common in these patients. Moreover, patients receiving complex spine surgery are mostly older in age, and may suffer from multiple comorbidities; therefore, the risks involved with anesthesia and surgery are increased in these patients. Our patient demographic data are compatible with this population, revealing a mean age of 65.53 ± 11.87 years with 85.7% of the participants belonging to the ASA class 3 category.

The values of perfusion index (PI) indicate the quality of recorded SpHb on the Radical-7™ Pulse CO-Oximeter monitor. Miller et al. [22] have reported a significant increase in the accuracy of the SpHb measurements when the PI was > 2.0 after receiving digital nerve block. Nguyen et al. [14] have documented an increase in Hb difference when the PI value was decreased. In our study, the statistical analyses revealed moderate correlations between SpHb and tHb in the two groups, with percentage errors (PE) of 15.85 and 17.42% in the < 1.0 and ≥ 1.0 groups, respectively. The clinically acceptable percentage error is considered < 30% [25, 26]. In the present study, the Radical-7™ Pulse CO-Oximeter monitor is able to demonstrate the acceptable accuracy in the hemoglobin concentration measurements when compared with the hemoglobin concentration measurements obtained using the blood gas analyzer. These findings are similar to those of a previous study reported by Miller et al. [19], wherein no significant difference between the PI < 1.4 and PI ≥1.4 groups was demonstrated. Although the mean absolute differences with PI values > 4.0 were smaller than those with PI value < 4.0, there were few measurements with PI value > 4.0. Our results are supported by the study of Berkow et al. who had evaluated the patients undergoing spine surgery [20]. Instead of using the perfusion index to separate the pairs of data, they adopted a signal-quality indicator (SIQ) for the SpHb values. The Bland–Altman plots revealed that the mean bias was − 0.3 g/dL with LoA ranging from − 2.4 to 1.7 g/dL and a PE ranging from 12.37 to 19.89%. For the data pairs excluding the low SIQ value, the mean bias was − 0.1 g/dL with LoA ranging from − 2.0 to 1.8 g/dL and a PE ranging from 10.22 to 17.28%. In that study, the median PI during low SIQ periods was approximately 0.5%. However, other studies have reported broader LoA compared with our results. Colquhoun et al. [23], who included patients undergoing major spine surgery, have reported the mean bias was − 1.27 g/dL with LoA ranging from − 5.05 to − 2.51 g/dL. Furthermore, the accuracy of SpHb was not positively correlated to the PI values [22]. Further studies determining the adequate cutoff of the perfusion index for interpretation of the SpHb data are warranted.

The results of the current study may not be satisfactory in terms of testing the trending ability. According to the study by Critchley et al., a concordance rate of > 92% is considered as good and acceptable for evaluation of the trending ability [25, 26]. In the present study, the concordance rates were 85.11% in the four-quadrant plot method and 67.21% in the Critchley polar plot method; both these results were < 92%. Huang, et al. [11] and Kayhan et al. [27] have reported the concordance rate of 87 and 79% in patients undergoing liver transplantation, respectively. Similarly, the concordance rate in another study was found to be below the acceptable limits [23].

Prone position is acquired in the posterior approach spine surgery to assess the dorsal side of the body. Increased intra-abdominal pressure in prone position contributes to subsequent physiological changes such as decreased cardiac index, inferior vena caval obstruction, and pulmonary ventilation/perfusion distribution [28]. It is reported that compared with the supine position, the prone position adversely affects the PI values [29]. In the present study, no significant differences in SpHb data were noted between the supine and prone positions in the same patient. To the best of our knowledge, there is no documented article revealing whether the change in position influences the accuracy of SpHb measurement.

In the present prospective observational study, we did not analyze the effect of Radical-7™ Pulse CO-Oximeter on transfusion therapy. From our perspective, we consider that the ability of this monitor to reduce over- and under-transfusion during surgery. Awada et al. have reported that fewer blood products were transfused and a shorter duration of delayed transfusion was observed in SpHb-guided group [15]. Another study has demonstrated a 4% absolute reduction in the risk of blood transfusion in the group monitored by SpHb, with no difference in the amount of RBC units transfused and 28-day complication rates between the two groups [21].

Several factors influence the peripheral blood perfusion such as use of vasopressors, body temperature, and severe anemia. The present study has some limitations. First, we did not encounter cases with significant hypothermia (body temperature < 35 °C) or severe anemia (Hb < 7 g/dL) or that of patients using vasopressors; so we were unable to test the accuracy of SpHb in such groups. Besides, although the SpHb sensor was covered to avoid optical interference, environment light pollution may be of concern in the accuracy of results. Furthermore, we did not compare the cost efficiency of the SpHb monitor with standard laboratory analysis, which may be of importance in several institutions worldwide. A larger sample size including diverse patient populations is recommended. Although we had divided the PI values into two groups for statistical analyses, the relation between PI and the monitor accuracy remains interesting and several levels of PI in different groups may be considered for analysis. As mentioned above, several factors can pose a challenge and further studies investigating these factors are required in future.

The traditional central laboratory analysis is time consuming and may not be available in some institutions, although the incorporation of point-of-care satellite laboratory stations in operating rooms has gained popularity. In addition, it is potentially risky for clinicians who may be injured or infected during withdrawal of blood samples every day. The Masimo Radical-7™ Pulse CO-Oximeter is a non-invasive, immediate, and consecutive method that perioperatively provides the hemoglobin concentrations of the patients. The use of non-invasive monitoring techniques is popular in clinical practice in the current era; nevertheless, this study does not advocate it as a replacement of the traditional methods of measurement. On the contrary, laboratory analyses continue to remain as the gold standard for the accumulation of accurate data. It is rational to check the laboratory values at critical time points when significant intraoperative events occur (e.g., surgical site bleeding or great vessel injury) or when remarkable variations in the monitor data are noted (e.g., sudden decrease in the SpHb value). The use of the monitoring device in conjunction with laboratory analysis may prove helpful in clinical practice.

## Conclusion

In conclusion, the present study demonstrates the acceptable accuracy of the Masimo Radical-7™ Pulse CO-Oximeter in measuring the hemoglobin concentration even under low perfusion index levels. Although the trending ability of this monitor is limited and unsatisfactory, it may be used as a reference for making decisions with regard to transfusion therapy for complex spine surgery.

## Data Availability

The datasets used during the current study are available from the corresponding author on reasonable request.

## Abbreviations

ASA: American Society of Anesthesiologists
BMI: Body mass index
FFP: Fresh frozen plasma
Hb: Hemoglobin
IV: Intravenous
LoA: Limits of agreement
PE: Percentage error
PI: Perfusion index
pRBC: Packed red blood cells
r: Correction coefficients
SD: Standard deviation
SpHb: Noninvasive and continuous hemoglobin concentration
tHb: Total hemoglobin concentration
